# Robbery in progress: Historical museum collections bring to light a mitochondrial capture within a bird species widespread across southern Australia, the Copperback Quail‐thrush *Cinclosoma clarum*

**DOI:** 10.1002/ece3.6403

**Published:** 2020-05-31

**Authors:** Kerensa McElroy, Andrew Black, Gaynor Dolman, Philippa Horton, Lynn Pedler, Catriona D. Campbell, Alex Drew, Leo Joseph

**Affiliations:** ^1^ Australian National Wildlife Collection CSIRO National Research Collections Australia Canberra ACT Australia; ^2^ South Australian Museum Adelaide SA Australia; ^3^ Molecular Systematics Unit Western Australian Museum WA Australia; ^4^ University of Adelaide Adelaide SA Australia; ^5^ Koolunga SA Australia

**Keywords:** *Cinclosoma*, mitochondrial capture, museum specimens, phylogeography, quail‐thrush, southern Australia

## Abstract

We surveyed mitochondrial, autosomal, and Z chromosome diversity within and between the Copperback Quail‐thrush *Cinclosoma clarum* and Chestnut Quail‐thrush *C. castanotum*, which together span the arid and semi‐arid zones of southern Australia, and primarily from specimens held in museum collections. We affirm the recent taxonomic separation of the two species and then focus on diversity within the more widespread of the two species, *C. clarum*. To guide further study of the system and what it offers to understanding the genomics of the differentiation and speciation processes, we develop and present a hypothesis to explain mitonuclear discordance that emerged in ourdata. Following a period of historical allopatry, secondary contact has resulted in an eastern mitochondrial genome replacing the western mitochondrial genome in western populations. This is predicted under a population‐level invasion in the opposite direction, that of the western population invading the range of the eastern one. Mitochondrial captures can be driven by neutral, demographic processes, or adaptive mechanisms, and we favor the hypothesized capture being driven by neutral means. We cannot fully reject the adaptive process but suggest how these alternatives may be further tested. We acknowledge an alternative hypothesis, which finds some support in phenotypic data published elsewhere, namely that outcomes of secondary contact have been more complex than our current genomic data suggest. Discriminating and reconciling these two alternative hypotheses, which may not be mutually exclusive, could be tested with closer sampling at levels of population, individual, and nucleotide than has so far been possible. This would be further aided by knowledge of the genetic basis to phenotypic variation described elsewhere.

## Graphical abstract

We present a case that within the Copperback Quail‐thrush *Cinclosoma clarum*, a widespread bird of southern Australia's arid and semi‐arid zones, a mitochondrial capture is taking place driven by neutral demographic processes. This in turn reflects a likely range expansion of the species' western populations into the range of its eastern populations. We can see the need for further analyses to test this hypothesis as well as an alternative and so guide the system's contribution to the genomic study of differentiation and speciation.

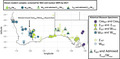

## 1 INTRODUCTION

Literature is growing rapidly on the genomics of speciation and introgression. A facet of this that we focus on here concerns mitochondrial DNA introgression and the mitonuclear discordance it can generate (Rheindt & Edwards, 2011; Winger & Bates, 2015; Roux et al., 2016; Bonnet, Leblois, Rousset, & Crochet, 2017; Gompert, Mandeville, & Buerkle, 2017; Ottenburghs et al., 2015; Ottenburghs et al., 2017;Joseph, 2018; Taylor & Larson, 2019; Peñalba, Joseph, & Moritz, 2019 and references in each). Two principal mechanisms are used to explain a particular form of mitonuclear discordance, that of the capture and replacement of one mitochondrial genome by another: adaptive, mitonuclear interactions, or neutral demographic processes accompanying a range invasion of one taxon or population by another (Currat, Ruedi, Petit, & Excoffier, 2008). Here we report a case of discordance between mitochondrial and nuclear data sets within a southern Australian bird species, the Copperback Quail‐thrush *Cinclosoma clarum* Morgan, 1926. Our aim is to propose a hypothesis that this is an active, ongoing mitochondrial capture driven by neutral demographic processes. We acknowledge some issues hindering unambiguous interpretation of our data, but we hope that presenting the hypothesis here will guide further study of this system.

Recently recognized as a species distinct from the Chestnut Quail‐thrush *C. castanotum* to its east (Dolman & Joseph, 2015, 2016; Toon, Austin, Dolman, Pedler, & Joseph, 2012), *C. clarum* is vastly distributed across southern Australia (Figure S1). Cryofrozen tissue samples for genetic analysis are still not available from much of the range of *C. clarum*. We have addressed this sampling gap here using historical museum collections and some newly collected material. After reporting the results of screening this material, we develop and present our hypothesis while framing our discussion around the current intraspecific taxonomy of *C. clarum* (Black, Joseph, Pedler, & Horton, 2019; Figure S1), which was based on diversity in plumage patterns. Three subspecies (*C. cl. clarum*, *C. cl. fordianum*, *C. cl. morgani*) and several zones of intergradation among them are currently recognized (details in Figure S1).

## 2 METHODS

**FIGURE 1:**
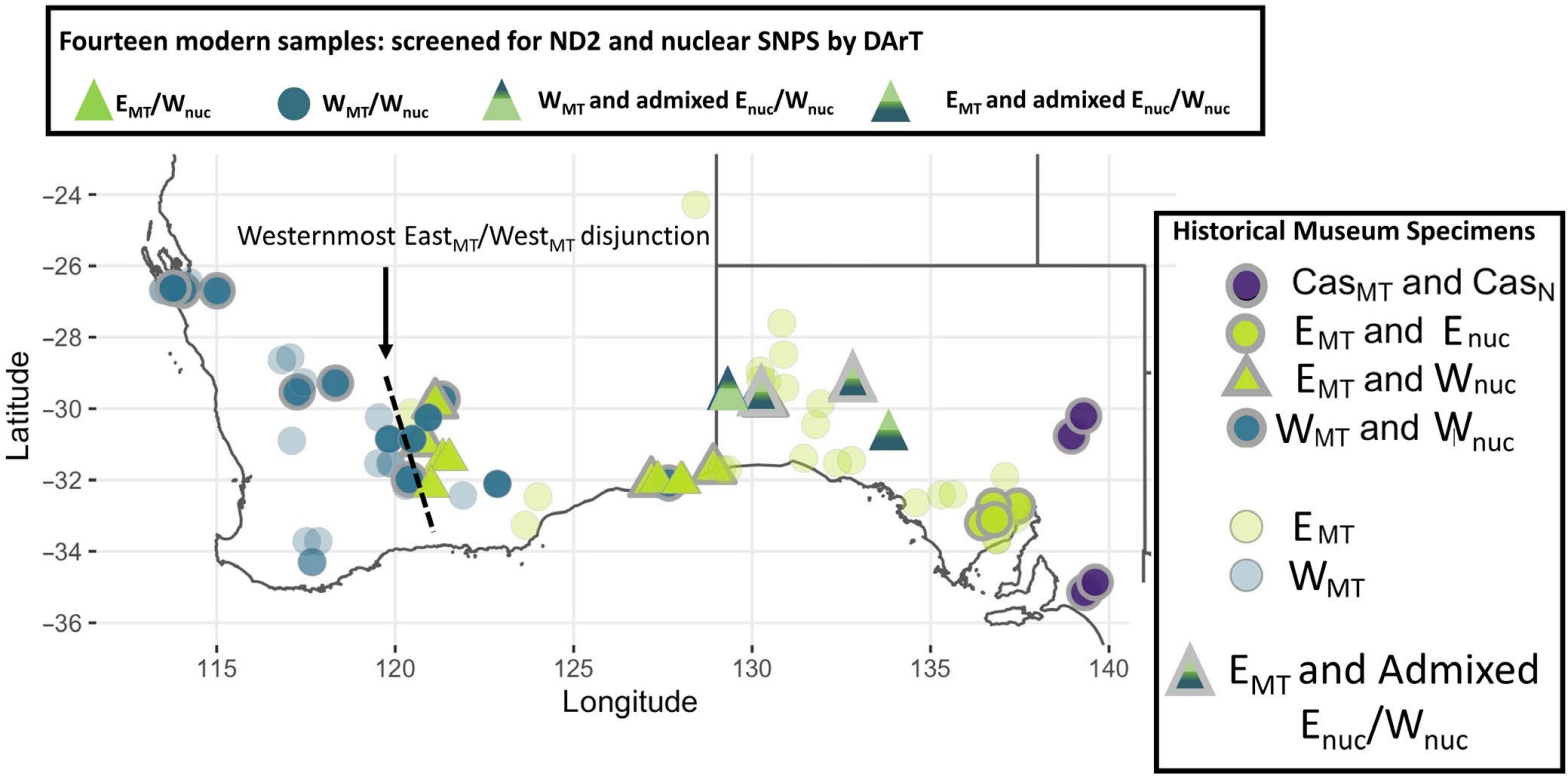
Map highlighting the two key observations concerning the eastern mitogenome of *C. clarum* (light green circles and triangles) in support of the hypothesis offered here: (1) it has been recorded deep into the western part of the species’ range to the dotted line, and (2) it occurs extensively in both eastern and western nuclear genomic backgrounds (symbols with heavy gray borders). Specimens estimated by STRUCTURE analysis (Figure 2) to have substantially admixed nuclear genomes (East_nuc_/West_nuc)_ are indicated by shaded symbols with heavy gray borders (SAMA B57995 and SAMA B55332). Similarly, the DArT analysis's (Figure S2) estimate of what we infer in the text to be substantially admixed nuclear genomes are indicated by shaded triangles without heavy gray borders (specimens ANWC B51857, ANWC B52266, ANWC B51855 and ANWC B52080. Abbreviations: E, East; MT, mitogenome; nuc, nuclear genome; W, West, respectively (e.g., East_MT_ of main text becomes E_MT_). The four *C. castanotum* specimens selected for SNP analysis are also indicated (purple circles with CAS abbreviation). Museum registration numbers of the 18 *C. clarum* and four *C. castanotum* with heavy gray borders are given in Table S2 and registration numbers of all specimens are linked with their mitochondrial haplotype in Appendix S1

Appendix S1 provides full details of DNA extraction, generation, and analysis of all molecular data, the main details being summarized here. Seventy‐two historical museum specimens distributed across the combined ranges of *C. castanotum* and *C. clarum* were selected from the collections of the Western Australian Museum (WAM), the South Australian Museum (SAM), and the Australian National Wildlife Collection (ANWC) for mitogenomic study and single nucleotide polymorphism (SNP) analysis from toe pads; single specimens of another species of *Cinclosoma*, the Spotted Quail‐thrush *C. punctatum* and from a species in the sister genus, the Blue‐backed jewel Babbler *Ptilorrhoa caerulescens,* were included as outgroups (Appendix S1). Males and females were included and dates of specimen collection ranged from 12 August 1902 to 2 April 2008 (Appendix S1). Figure 1 shows locations of the 72 specimens and data from them can be found in metadata available at https://doi.org/10.25919/5b70dc3a7ecf7. DNA was extracted from the historical museum specimens from 2 mm^3^ toe pad slices, using the protocol described in McElroy, Beattie, Symonds, and Joseph (2018). Informed heavily by Dolman and Joseph’s (2015, 2016) geographical sampling gaps especially for the west of the species’ range, we also selected a set of 18 *C. clarum* specimens (asterisked in Appendix S1) and four *C. castanotum* for further whole genome sequencing with which to generate a mitochondrial, autosomal, and Z chromosome dataset. The four *C. castanotum* were included to further assess the nature of any genetic disjunction between *C. clarum* and *C. castanotum*. Localities of these 22 specimens are marked by thick gray borders in Figure 1 (see also Appendix S1).

Genomic SNPs were called for specimens sequenced to higher coverage (Appendix S1). The GATK pipeline v3.5‐0‐g36282e4 (McKenna et al., 2010) was used to call SNPs from these alignments. SNPs were filtered to exclude the mitochondrial genome and as follows: “QD < 2.0 || FS > 60.0 || MQ < 40.0 || MQRankSum < ‐12.5 || SOR > 4.0 || ReadPosRankSum < ‐8.0,” resulting in a total of 60,966,750 nuclear SNPs. The clustering program Structure v2.3.4 (Pritchard, Stephens, & Donnelly, 2000) was used to assess subdivision of *Cinclosoma* populations based on autosomal SNPs. A separate Structure run was performed for the Z chromosome, using the same filtered set of 60,966,750 SNPs, but restricted to the Z chromosome.

Ten more *C. clarum* specimens with cryofrozen tissue samples became available as the study progressed. They came from ANWC field work in south‐eastern Western Australia in 2017 (Figure 1; Appendix S1) after the first phase of work just described had been completed. Critically, they spanned the region in the west of the species’ range for which historical museum specimens provided more thorough geographical coverage. They thus provided an independent check on the validity of data from the older specimens. Using DNA extracted from cryofrozen liver samples held in the ANWC and together with an eleventh specimen, ANWC B51857, which showed mitonuclear discordance in Dolman and Joseph’s (2015, 2016) study, they were directly sequenced for mitochondrial DNA (ND2) and assayed for nuclear SNPs using DNA provided to Diversity Arrays Technology, Canberra (DArT) with a concentration of 30 ng/µl. This analysis also used three specimens collected from the same part of the range as B51857 and four *C. castanotum*. Extraction was done using the Qiagen Puregene^®^ Tissue Kit following the manufacturer's protocols.

The R package dartR (Gruber, Unmack, Berry, & Georges, 2018) was used for population genomic analyses of the DArTseq SNP data, including filtering data and principal coordinate analysis (PCoA). For mitogenome reconstruction, quality assessment and trimming of raw reads were performed exactly as described in (McElroy et al., 2018). For mitochondrial phylogenetic analysis, forward and reverse raw sequences were aligned in Geneious 10.2 (Biomatters) and subsequently checked and edited manually. The consensus sequence for all individuals was aligned in MUSCLE (Edgar, 2004). The Hasegawa–Kishino–Yano (HKY; Hasegawa, Kishino, & Yano, 1985) model was chosen with percentage of invariant sites using AIC calculations in MEGA X v10.0.5 (Kumar, Stecher, Li, Knyaz, & Tamura, 2018). Phylogenetic trees were prepared using Garli v2.01 (Zwickl, 2006).

To provide a visual overview of all datasets on one map, location, sequencing, and genotype data from previously described analyses were integrated into a single tidy data frame using the R tidyverse_1.2.1 library (Wickham, 2017). Map and state boundaries were drawn using sf_0.8‐0 (Pebesma, 2018), with points placed using the tidyverse's ggplot tools.

## 3 RESULTS

### 3.1 Mitochondrial reconstruction

Genomic data have been lodged at https://doi.org/10.25919/5b70dc3a7ecf7. Mitochondrial genomes were successfully reconstructed for 68 (i.e., 62 *C. clarum*; 4 *C. castanotum*; 1 *C. punctatum*; 1 *Ptilorrhoa caeruelescens*) historical museum specimens. Reconstructions covered an estimated 98% of the complete mitochondrial genome, based on comparison with the reference *T. guttata* mitochondrial genome. Within this reconstructed region, completion varied between 82% and 100%. Appendix S1 gives the length of the reconstructed region and the percentage of reconstructed bases for each specimen; it also details why three further specimens were excluded from analyses.

### 3.2 Mitogenome phylogeny, diversity

ND2 sequences were either extracted from the full mitogenome data or sequenced afresh from the 10 specimens collected in 2017 (GenBank accession numbers MT296788–MT296797). The 72 in‐group specimens (62 museum specimens + 10 from 2017) from which data were recovered fell into two clades (Appendix S1), which we refer to as East_MT_ and West_MT_. Figure 1 summarizes the geographical distribution of these two clades. Table 1 summarizes diversity statistics measured from ND2 or with whole mitogenomes. The net divergence in ND2 between *C. castanotum* and *C. clarum* (4.38%; Dolman & Joseph, 2015) was confirmed in this study at 4.5%, whereas in the whole mitogenome data it was higher at 6.7%. Within *C. clarum*, the East_MT_ and West_MT_ subclades have a net divergence from each other of *ca* 1.50% (ND2) and 1.40% (whole mitogenomes) and *F*_ST_ between them is high at 0.693 and 0.102, respectively.

**TABLE 1:** Summary of population diversity statistics measured from ND2 mtDNA sequence data or whole mitogenomes

Data set	Statistic	Comparison	
		*C. castanotum* vs.* C. clarum*	East_MT_ vs. West_MT_ within *C. clarum*
ND2	*F*_ST_	0.842	0.693
	*D_xy_*	0.045	0.015
	*D*_a_	0.045	0.015
Whole mitogenomes	*F*_ST_	0.489	0.102
	*D_xy_*	0.138	0.141
	*D*_a_	0.067	0.014

### 3.3 Nuclear structure

**FIGURE 2:**
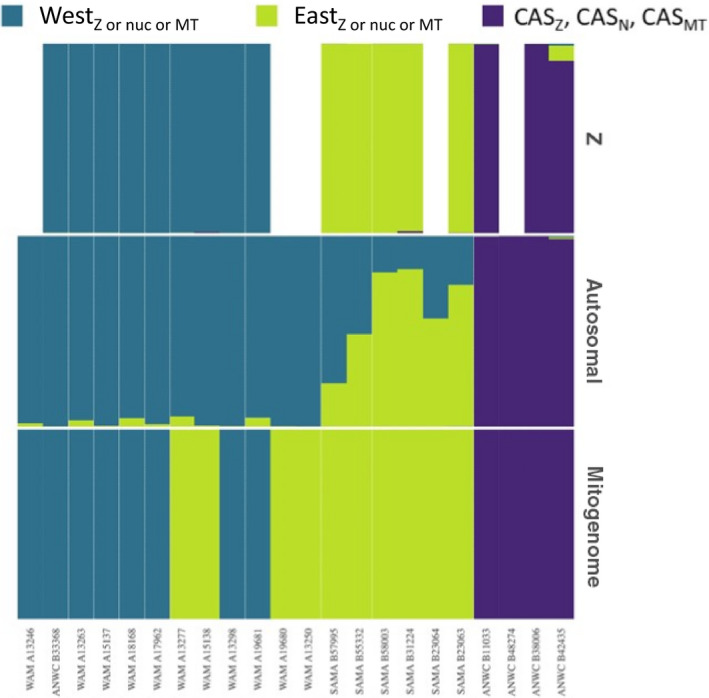
Results of STRUCTURE analysis for Z chromosome, autosomal, and mitogenome data arguing *k* = 3 as the optimal number of populations based on all 12 runs of the analysis involving 18 *C. clarum* (shades of green) and four *C. castanotum* (purple) and corresponding to all those symbols with heavy gray borders in Figure 1. Blank columns indicate no data for the individual in that category. Specimens assayed are arranged left to right in geographical (west to east) sequence. Museum registration number of each specimen is indicated at the base of their respective columns. ANWC, Australian National Wildlife Collection; SAMA, South Australian Museum; WAM, Western Australian Museum. Shorthand for Z chromosome variation follows that for mitogenomes and autosomal genomes in the main text that is, West_Z_, and East_Z_ within *C. clarum*. CAS—*C. castanotum*. The Z, nuclear autosomal, and mitochondrial genomes of *C. castanotum* are CAS_Z_, CAS_N_, and CAS_MT_, respectively

**FIGURE 3:**
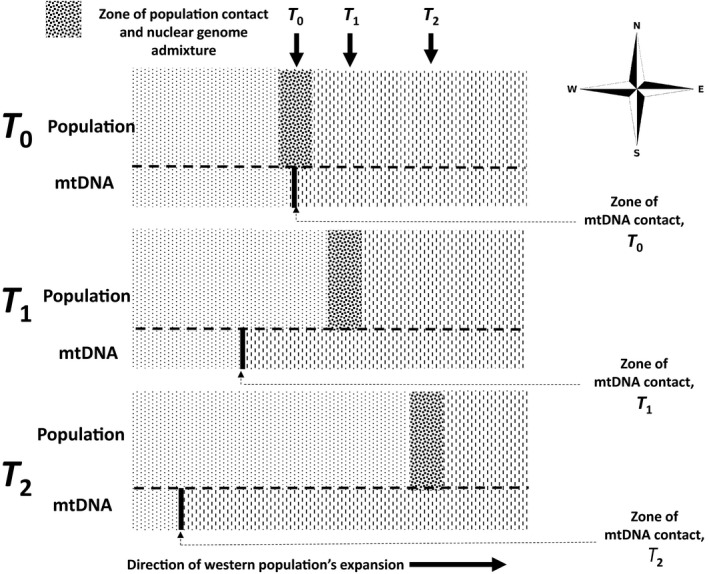
Schematic illustration of the demographic process of mitochondrial capture described in the text. Western populations expand their range eastwards into the range of the eastern populations. The mitochondrial genome of the “invaded” eastern populations (in this case mainly represented by *C. clarum clarum* and *C. clarum morgani*) extensively introgresses westwards into the nuclear genomic background of the “invader” western populations (in this case mainly represented by *C. clarum fordianum*). *T*_0_ is the time of initial contact between western and eastern populations as western populations expand into the range of eastern populations. *T*_1_ and *T*_2_ are successively later stages of the western populations’ expansion. See the text for further details of the present case

Alignment of sequence reads from the 22 specimens selected for mitochondrial, autosomal, and Z chromosome analysis specimens to the *T. guttata* genome was successful, yielding a mean read depth of 17,323,937 to 109,118,947 bp, and low read duplication levels of between 0.28 and 4.10 (Appendix S1). 89,619,382 SNP calls were obtained from these alignments, which were further filtered to produce a set of 6,125 genome‐wide nuclear markers (SNPs) for a Structure clustering analysis. Figure 3 shows the consensus results for 12 independent STRUCTURE runs, assuming (a) two, (b) three, and (c) four underlying populations. The *Evanno* method agreed with our visual interpretation, identifying three populations as the best model for our data. One of these corresponds to the species *C. castanotum* (purple in Figure 2) and the other two (shades of green in Figure 2) are within our focal species here, *C. clarum*. Therefore, and as with labeling of mitogenomes for ease of discussion, we refer to these two categories of autosomal parts of the nuclear genomes within *C. clarum* as East_nuc_ and West_nuc_.

Notably, there is no evidence of interspecies admixture between *C. castanotum* and *C. clarum*. Nuclear population assignment within the species *C. clarum*, however, is strikingly discordant with the mitogenomic assignment of individuals to the East_MT_ or West_MT_ mtDNA clades, which is indicated on the horizontal axis of Figure 2. For example, the five westernmost of the 11 East_MT_ individuals are in all or predominantly West_nuc_ nuclear backgrounds (green triangles) and so largely typical of western populations (Figure 1). Evidence for substantial admixture (i.e., two shades of green in Figure 2) is most evident in the geographically most central and eastern of the 18 specimens.

### 3.4 Specimens collected in 2017

The DArTseq approach on cryofrozen material generated 70,627 SNPs. After filtering to remove loci with missing data, 16,602 SNPs were retained for further analyses. PCoA1 (Figure S2) clearly separates the four *C. castanotum* from the 14 *C. clarum* (10 specimens from 2017 from the western part of the range and four from the geographical center and east of the range). All are therefore assigned as expected to their respective species.

On PCoA2, however, the ten 2017 specimens of *C. clarum* cluster tightly in Figure S2’s top right quadrant but well apart from the four more central and eastern *C. clarum* samples in the lower right quadrant. These 10 specimens from 2017 are from the same region as the STRUCTURE analysis's West_nuc_. They are from zones currently considered to be the range of either subspecies *C. cl. fordianum* or phenotypic intergrades between *C. cl. clarum* and *C. cl. fordianum* (Figure S1). We therefore infer this to be the DArT method's detection of STRUCTURE’s (Figure 2) West_nuc_ genome.

The four *C. clarum* specimens in Figure S2’s lower right quadrant are from the geographical region that STRUCTURE (Figure 2) estimated to be admixed West_nuc_/East_nuc_. This corresponds well with them coming from a zone of intergradation between subspecies *C. cl. clarum* and *C. cl. fordianum* (Figure S1). We infer this to be the DArT method's detection of the same admixture.

Lastly, the latter cluster includes ANWC B51857, which was previously (Dolman & Joseph, 2015) shown to be an anomalously eastern occurrence of West_MT_. In Appendix S1, it clearly aligns with geographically close admixed West_nuc_/East_nuc_ specimens. We infer that it is indeed the only sample in our survey having West_MT_ in a nuclear background that it is not solely West_nuc_.

Figure 2 incorporates all of these results.

## 4 DISCUSSION

Three key findings emerged from our survey of mitochondrial and nuclear diversity within and between the Chestnut Quail‐thrush *Cinclosoma castanotum* and the Copperback Quail‐thrush *C. clarum*. First, we robustly affirmed their recent taxonomic separation as two species (Dolman & Joseph, 2015, 2016). Second, we described a broad pattern of simple east–west geographic structure in genetic diversity within *C. clarum*; admixture is most evident in nuclear genomes of samples from the geographical center and, to a lesser extent, the east of the range. This suggests a period of allopatric differentiation during which eastern and western subgroups evolved within the species. Simple secondary contact in the geographical center of the species’ range explains this pattern of admixture. We suggest, however, that it does not explain the third key set of inter‐related findings centered on the eastern mitochondrial genome: *C. clarum's* eastern mitochondrial genome is found in individuals that have the species’ eastern nuclear genomic background; that same eastern mitochondrial genome also extends geographically deep into the species’ overall western range; there it is also found in individuals having the western nuclear genomic background; lastly, the eastern mitochondrial genome also occurs in the individuals in the geographical center of the range having admixed nuclear backgrounds. The reciprocal pattern (western mitochondrial genome in eastern nuclear background), given our sampling, has not been observed. One individual having the western mitochondrial genome (ANWC B51857) was inferred to be in the admixed nuclear genomic background. It is from a zone which all other phenotypic (Black et al., 2019) and genotypic data (this study) suggest to be a simple zone of admixture and secondary contact. We now propose a hypothesis to guide further study of this system as a model of understanding the genomics of differentiation and speciation.

We propose the hypothesis that we have observed a “robbery in progress”: The western populations of *C. clarum* are in the process of having their mitochondrial genome replaced, or captured, by the mitochondrial genome of the eastern populations. Either of two processes can underpin this hypothesis. First is a neutral, demographically driven process (Figure 3). Here this would be that the western *population* generated in earlier allopatry (and taxonomically represented most simply by the *C. cl. fordianum* subspecies) has invaded the range of what is essentially the eastern *population* generated by that allopatry. These are represented taxonomically now most simply by what Black et al. (2019) distinguished as the two subspecies *C. cl. clarum* and *C. cl. morgani*. Introgression of mitochondrial genomes is then predicted to be in a direction opposite to that of the population invasion. That is, mitochondrial genomes would introgress from the eastern (invadee) into the western (invader; Currat et al., 2008; Drovetski et al., 2015; Petit & Excoffier, 2009; Toews & Brelsford, 2012). The nonexpanding eastern populations do not have the dynamics of fragmentation that the western population's invading front would be expected to have. This allows alleles of the western population at its invasion front to drift to fixation as it expands. An eastern population allele, once introgressed into the western population, however, can increase in frequency in western populations. This would happen through the dynamics of drift that western populations experience at their invasion front. A result is an mtDNA disjunction or mtDNA‐defined zone of contact extending deeper and deeper into the western population's *current* geographical range (see examples in Seixas, Boursot, & Melo‐Ferreira, 2018; Zhang et al., 2019). Here, this mtDNA disjunction is therefore expected to be west of the current zones of admixture as measured primarily in nuclear DNA characters (Figure 2) but also in phenotypic characters (Black et al., 2019; Figure 1). This is what we have observed (Figures 1 and 2). Figure 3 illustrates this schematically. Further, we expect geographical patterns of diversity in Z chromosome, autosomal, and mtDNA markers all to differ under this selectively neutral scenario, (e.g., Cortés‐Ortiz et al., 2019), and again this is what we have observed (Figure 2).

The second process that might underpin the hypothesis is adaptive. One would posit that the eastern population's mitogenome has acquired a selective advantage relative to the western population's mitogenome. This would happen when the former is introduced by introgression into the western population's nuclear genomic background. The eastern population's mitogenome could then have adaptively introgressed westwards into the original range of the western population. This would again imply that we have observed a mitochondrial capture still in progress. By this process, however, adaptively favorable combinations of autosomal and mitochondrial markers are expected to be inherited together (Hill, 2019). We would expect to observe at least some similar geographic patterns in autosomal and mtDNA markers. Our data (Figure 2) clearly do not show such similarity. We acknowledge that discriminating among the two explanations requires closer and finer sampling of nuclear genomes than we have done. This applies especially in the remote and difficult‐to‐access west of the species’ range. We cannot reject this adaptive mechanism completely. It should be pursued.

An alternative hypothesis, though not necessarily mutually exclusive, is that *C. clarum* has a much broader zone of secondary contact than our data suggest. This hypothesis predicts that closer sampling of the species’ range would reveal more admixture of the eastern and western nuclear genomes. Specifically, it predicts that the apparent rarity of the eastern nuclear genome in the west of the range, and that of the western mitochondrial genome in the east of the range, are both artifacts of inadequate sampling. The clear predominance almost to the point of uniformity of the western nuclear genome in Black et al.'s (2019) hybrid swarm zone (Figure S1), which they defined primarily on dorsal plumage variability (e.g., their page 19), was particularly unexpected. Two explanations may reconcile this and other discrepancies between genotypic and phenotypic patterns given our hypothesis of a mitochondrial capture. First, it is feasible that dorsal plumage is under the control of very few genes (e.g., Toews et al., 2016). If so, then the very low frequency of the eastern nuclear genomic signature in the geographically westernmost samples (Figure 2) is unsurprising. Second, strong selection may lead to consistent plumage patterns in some habitats (e.g., arid core range of *C. cl. clarum*). Conversely, its relaxation may lead to high variability in other habitats (all regions west of *C. cl. clarum*). Supporting this speculation, we note that quail‐thrush are primarily terrestrial birds. Different dorsal plumages of the various species in the genus, not just in populations of *C. clarum*, are vital for their crypsis against different substrates from rainforests to deserts. Further work might test whether relative consistency of dorsal plumage patterning in *C. clarum* reflects differing strengths of selection against different substrates. Similar alternatives may be considered in explaining other discordances between phenotypic and genomic patterns.

Three possible limitations warrant discussion. First, it may be that the temporal spread of our samples renders it artifactual to analyze our data together as spatio‐temporally homogeneous. We counter with two points. Substantial zones of *concordance* involve specimens spanning a century or more, for example, the western populations and the range of *C. clarum morgani*. And these specimens represent a very short temporal window in the species’ evolutionary history, notwithstanding their critical role in completing spatial sampling.

A related second concern might be that of error rates in data from older historical specimens. Most of the historical specimens we used came from the 1960s or later, only 11 coming from the 1920s or earlier, and none from the 1930s to 1950s (Appendix S1). The part of the species’ geographical range that the historical specimens filled was nonetheless also spanned, albeit less thoroughly, by cryofrozen specimens collected between 2002 and 2017. Consistency of patterns across the different specimen types and the different methods by which they were analyzed negates this concern (see also Appendix S1: Detailed Methods). Further, several studies (Crates et al., 2019; Ewart et al., 2019 and references therein) also showed that when many more recent specimens are included, the level of error or failure of older specimens need not negate cautious interpretation of spatial patterns, which are our focus here (see also Billerman & Walsh, 2019).

A final limitation may be that we have not sampled the core range of *C. cl. clarum* sensu Black et al. (2019). This happened because Black et al.’s (2019) phenotypic analyses and this study's genomic analyses largely progressed in parallel until their respective later stages. Both built on earlier literature especially Ford (1981, 1983) and Schodde and Mason (1999). Samples thought at the outset of this genomic study to be from within the core range of *C. cl. clarum*, for example, were eventually assigned by Black et al. (2019) on phenotypic grounds to a *C. cl. clarum x C. cl. fordianum* zone of intergradation. Factors such as the remote core range of *C. cl. clarum* prevented more genomic data being obtained. Given that we see no hint of a third nuclear genomic signature within the species (Figure 2), we consider this a negligible limitation.

To summarize, we favor the hypothesis that a mitochondrial capture driven by neutral demographic processes is occurring in *C. clarum*. Although we cannot fully reject the alternative adaptive process, discriminating between the two processes ultimately requires that one test for an adaptive advantage of the eastern mitogenome in the western nuclear background. Population genetics‐based tests such as dN/dS ratios are a cautious starting point here (but see Kryazhimskiy & Plotkin, 2008). Nontrivial cellular level physiological work to test for mitonuclear coadaptation (e.g., Toews, Mandic, Richards, & Irwin, 2013; see also Hill, 2019) would ultimately be necessary. Perhaps more significant, however, it is intuitively unlikely that we should be able to observe an adaptively driven mtDNA capture in progress: a selective advantage may be expected to have already resulted in complete capture long before modern study of these birds. A neutral, demographic mechanism could have been initiated far more recently by climatic and environmental change since the Last Glacial Maximum, for example.

Further work should also explore linkages among geographic patterns in genotype, phenotype, and habitat. Does dorsal plumage patterning, which is so vital for the birds’ crypsis, change with substrate? Specifically, sampling is needed of the subspecies *C. clarum clarum* for comparison with more variable populations further west (Figure S1). Whether any such linkages are correlative or causative would inform the taxonomic significance, or otherwise, of these patterns. A second focus could be closer attention to drivers of Z chromosome variation. Recently, sometimes unexpected complexity of sex chromosome markers has been highlighted (*cf*. Lasne, van Heerwaarden, Sgrò, & Connallon, 2018; Battey, 2020; Hayes, Barton, & Zeng, 2020). This system offers much to the study of the genomics of differentiation and speciation.

## CONFLICT OF INTEREST

The authors declare no conflicts of interest.

## AUTHOR CONTRIBUTION

**Kerensa McElroy:** Data curation (lead); Formal analysis (lead); Methodology (lead); Visualization (lead); Writing‐original draft (supporting). **Andrew Black:** Conceptualization (supporting); Visualization (supporting); Writing‐review & editing (supporting). **Gaynor Dolman:** Formal analysis (supporting). **Philippa Horton:** Conceptualization (supporting); Writing‐review & editing (supporting). **Lynn Pedler:** Investigation (supporting). **Catriona D. Campbell:** Data curation (supporting); Formal analysis (supporting); Software (supporting). **Alex Drew:** Investigation (supporting). **Leo Joseph:** Conceptualization (lead); Visualization (supporting); Writing‐original draft (lead); Writing‐review & editing (lead).

## Supporting information

Appendix S1

## Data Availability

DNA sequences and whole mitogenomes newly acquired here have been lodged in GenBank with accession numbers MT296788–MT296797 and MT425442–MT425506. DaRT data are at https://doi.org/10.6084/m9.figshare.12177327. All other data are lodged in CSIRO’s public data depository at https://doi.org/10.25919/5b70dc3a7ecf7.
