# Supplementary material for: Robbery in progress: Historical museum collections bring to light a mitochondrial capture within a bird species widespread across southern Australia, the Copperback Quail‐thrush Cinclosoma clarum
Source: Ecol Evol. 2020 May 31;10(13):6785–93. doi: 10.1002/ece3.6403 (PMC7381587; doi:10.1002/ece3.6403)
Supplement: Supplementary file 1 — Appendix S1 [file ECE3-10-6785-s001.docx]

**ELECTRONIC SUPPLEMENTARY MATERIAL/APPENDIX**

**DETAILED METHODS**

To avoid contamination, all extractions were performed in a UV-sterilised laminar flow cabinet. Individually indexed paired-end Illumina sequence libraries were constructed for each DNA extract, using a modified version of [Meyer & Kircher’s (2010)](https://paperpile.com/c/ak8Pu0/pQNR) protocol as described in [McElroy *et al.* (2018)](https://paperpile.com/c/ak8Pu0/qn5W). This included minimising the effects of cytosine to uracil conversion (from age-related deamination) on downstream analysis by including 1.5 units of NEB USER enzyme (GeneSearch Pty Ltd, QLD, Australia) during blunt end repair before addition of T4 DNA polymerase and incubation for 3 h at 37°C (Briggs et al. 2010). To generate shallow ‘genome skimming’ data, suitable for mitochondrial genome reconstruction, indexed libraries were combined into a single equimolar pool prior to sequencing. A single lane of Illumina HiSeq 2500 high-throughput sequencing using v4.0 chemistry was performed by the Kinghorn Centre for Clinical Genomics (Garvan Institute of Medical Research, Sydney), yielding a mean of 3.5M reads per specimen for 65 specimens. To enable genome-wide analysis, and add some key specimens, a further four lanes of sequence data were generated for a subset of 17 original specimens and an additional seven specimens (four *castanotum*, one *clarum* and two outgroup taxa; mean total reads 104.5M each). These specimens were selected after initial mitochondrial and morphological analysis, to allow assessment of suspected mitonuclear discordance. Figure 1 (main text) indicates the locations and sequencing effort of all specimens. All raw sequence data has been deposited in CSIRO’s Data Access Portal (<https://doi.org/10.25919/5b70dc3a7ecf7>), including details of indexes, sequence runs, and raw read counts.

The DArTseq™ genotype-by-sequencing approach uses a combination of DArT complexity reduction and high throughput sequencing (Killian *et al.,* 2012; Courtois *et al*., 2013; Cruz *et al*., 2013) to simultaneously identify and genotype SNP markers in the absence of a reference genome. A combination of two enzymes was used for complexity reduction (*Pst*I-*Hpa*II), before addition of custom adapters (Killian *et al*., 2012) to restriction site overhangs. Fragments were amplified using primers complementary to the adapters. These also incorporated molecular identifier barcode tags, to allow multiplexing of up to 96 samples per sequencing run. PCR conditions consisted of: denaturation at 94°C for 1 minute; 30 cycles of 94°C for 20 seconds, 58°C for 30 seconds and 72°C for 45 seconds; and a final extension period of 72°C for 7 minutes. PCR products were pooled for sequencing on an Illumina HiSeq 2000 platform using 77 cycles of single end sequencing. Raw sequence reads were filtered and processed using a proprietary DArT analytical pipeline. Poor quality sequences were removed, with more stringent criteria being placed upon the barcode region than the rest of the sequence. Approximately 2,000,000 sequences per barcode were identified and used in marker calling, during which identical sequences were collapsed and filtered before screening to identify variable markers using DArT proprietary SNP and SilicoDArT algorithms (DArTsoft14). Similarly, we screened the ten specimens from 2017 for ND2 diversity to determine whether they were East_MT_ or West_MT_ using protocols of Dolman & Joseph (2015).

Pairwise distances were computed based on the Hamming distance, where the numbers of positions along the sequence which are different are compared (He *et al*. 2004). SNPs were filtered using the callrate function (95%) and the repavg function (99.5%). No monomorphic loci and no duplicate loci per sequence tag were identified.

Mitogenomes were reconstructed as described in [McElroy *et al.* (2018)](https://paperpile.com/c/ak8Pu0/qn5W) from trimmed reads using the program MITObim v1.8 [(Hahn *et al*. 2013)](https://paperpile.com/c/ak8Pu0/FbE1), read numbers for each specimen being capped at the first 3750000 read pairs. Initial reconstructions were performed against the Zebra Finch *Taeniopygia guttata* mitochondrial reference genome [(Warren *et al.,* 2010)](https://paperpile.com/c/ak8Pu0/cALV), from release 3.2.4 (accession NC_007897.1). For some specimens, reconstruction was repeated with respect to the reconstructed mitogenome of one specimen ANWC B55864, in order to improve reconstruction completion by using a more closely related reference. For some reconstructions, where visual analysis of the alignment indicated manual correction of the reconstruction may be of assistance, this was also performed. Appendix Table 2 contains details of final reconstruction alignment coverages and qualities.

Trimmed reads were first aligned to the complete *T. guttata* genome [(Warren *et al.* 2010;](https://paperpile.com/c/ak8Pu0/cALV) refseq accession GCF_000151805.1) using the ‘mem’ algorithm from BWA 0.7.12-r1039 ([Li and Durbin 2009)](https://paperpile.com/c/ak8Pu0/C0mz), an initial match length of 19, an alignment score threshold of 30, indel bandwidth 50, and internal seed threshold of 1.3. Picard v1.138 was used on merged bam files for each specimen, to generate alignment quality statistics and to mark optical and PCR duplicates.

Briefly, indel realignment was performed per specimen using IndelRealigner, with per-interval coverage downsampled to 1000 (to speed up realignment in repetitive genomic regions, which are ultimately discarded). HaplotypeCaller was then applied in GVCF mode, before final, combined genotype calling with GenotypeGVCFs. Base quality score recalibration was not performed, due to insufficient data (based on GATK’s recommendations).

Due to lack of appropriate validation datasets (typically only available for model organisms), variant quality score recalibration was also not performed. Instead, raw variant calls were hard filtered with VariantFiltration.

To generate independent, genome-wide, informative SNP markers for this analysis, the 60966750SNPs attained above were further filtered using VCFtools v0.1.15, selecting only those autosomal SNPs that had a minor allele count of 3 or greater (to ensure polymorphic sites), a maximum mean depth of 13 (to exclude erroneous high coverage alignment in repetitive regions; mean read depth across sites and specimens is 6.7, having standard deviation 3.0, cutoff calculated as mean + 1.96 x SD), and no more than five missing genotypes. No minimum per-site read depth filter was applied; instead, we rely on GATK’s sophisticated statistical analysis to reject poor quality SNP calls. Finally, SNPs were thinned to be at least 200,000 base pairs apart, resulting in a final set of 5770 SNP markers across the autosomal genome. Structure was run with the number of repetitions set to 500000 and a burn in of 50000, with no location prior set. One, two, three, and four populations were tested, with twelve individual Structure runs for each (48 runs in total).

We further filtered this set to include only male specimens (females only have one Z chromosome, complicating the analysis), selecting SNPs with a minor allele count of 3 or greater (to ensure polymorphic sites), a maximum mean depth of 10 (mean read depth across male specimens along the Z chromosome is 5.4, with standard deviation 2.2, with cutoff calculated as mean ± 1.96 x SD), and no more than 4 missing genotypes.

Structure results were summarised using CLUMPAK [(Kopelman *et al.* 2015)](https://paperpile.com/c/ak8Pu0/qO4D) and visualised using the R package pophelper v2.0.0 [(Francis, 2017; R Core Team, 2018)](https://paperpile.com/c/ak8Pu0/bjbn). For visualisation, mitogenome assignment was manually coded in an appropriate format, to allow a multipanel pophelper image showing aligned Z, mitochondrial, and autosomal genotypes for each specimen.

**Additional References Cited Here**

Briggs, A. W., Stenzel, U., Meyer, M., Krause, J., Kircher, M., & Pääbo, S. (2010). Removal of deaminated cytosines and detection of *in vivo* methylation in ancient DNA. *Nucleic Acids Research,* *38,* e87. doi:10.1093/nar/gkp1163

Courtois, B., Audebert, A., Dardou, A., Roques, S., Ghneim- Herrera, T., Droc, G.,Frouin, J., Rouan, L., Gozé, E., Kilian, A., Ahmadi, N &. Dingkuhn, M. (2013). Genome-wide association mapping of root traits in a japonica rice panel. *PLoS ONE, 8,* e78037. doi:10.1371/journal.pone.0078037.

Cruz, V.M.V., Kilian, A., & Dierig, D.A. (2013). Development of DArT marker platforms and genetic diversity assessment of the US collection of the new oilseed crop lesquerella and related species. *PLoS ONE, 8,* e64062.

[Francis, R. M. (2017). Pophelper: an r package and web app to analyse and visualize population structure. *Molecular Ecology Resources*](http://paperpile.com/b/ak8Pu0/bjbn)*,* [*17,* 27–32.](http://paperpile.com/b/ak8Pu0/bjbn)

[Hahn, C., Bachmann, L., & Chevreux, B. (2013). Reconstructing mitochondrial genomes directly from genomic next-generation sequencing reads—a baiting and iterative mapping approach. *Nucleic Acids Research*](http://paperpile.com/b/ak8Pu0/FbE1)*,* [*41(13),* e129.](http://paperpile.com/b/ak8Pu0/FbE1) doi: 10.1093/nar/gkt371.

He, M.X., Petoukhov, S.V., & Ricci, P.E. (2004). Genetic code, hamming distance and stochastic matrices. *Bulletin of Mathematical Biology, 66*, 1405-1421.

Killian, A., Wenzl, P., Huttner, E., Carling, J., Xia, L., Blois, H., Caig, V., Heller-Uszynska, K., Jaccoud, D., Hopper, C., Aschenbrenner-Kilian, M., et al. (2012). Diversity arrays technology: a generic genome profiling technology on open platforms. *Methods in Molecular Biology, 888,* 67–89.

[Kopelman, N.M., Mayzel, J., Jakobsson, M., Rosenberg, N.A., & Mayrose, I. (2015). Clumpak: a program for identifying clustering modes and packaging population structure inferences across K. *Molecular Ecology Resources*](http://paperpile.com/b/ak8Pu0/qO4D)*,* [*15,* 1179–91.](http://paperpile.com/b/ak8Pu0/qO4D)

[Meyer, M., & Kircher, M. (2010). Illumina sequencing library preparation for highly multiplexed target capture and sequencing. *Cold Spring Harbor Protocols* 2010(6):pdb.prot5448. doi: 10.1101/pdb.prot5448.](http://paperpile.com/b/ak8Pu0/pQNR)

R Core Team (2018). R: A language and environment for statistical computing. R Foundation for Statistical Computing, Vienna, Austria. URL https://www.R-project.org/.

[Warren, W.C., Clayton, D.F., Ellegren, H., Arnold, A.P., Hillier, L.W., Künstner, A., Searle, S. *et al*. 2010. The genome of a songbird. *Nature*](http://paperpile.com/b/ak8Pu0/cALV)*,* [*464,* 757–62.](http://paperpile.com/b/ak8Pu0/cALV)

**APPENDIX FIGURES OR SUPPLEMENTARY MATERIAL FIGURES**

**Appendix Figure 1.** Map showing intraspecific taxonomy and geographical distribution of *Cinclosoma clarum sensu* Black *et al.* (2019); reproduced with permission. Key geographic regions mentioned in the text are shown. Abbreviations: NT – Northern Territory; SA – South Australia; WA – Western Australia. The separate species *C. castanotum* occurs east of the map’s eastern limit.


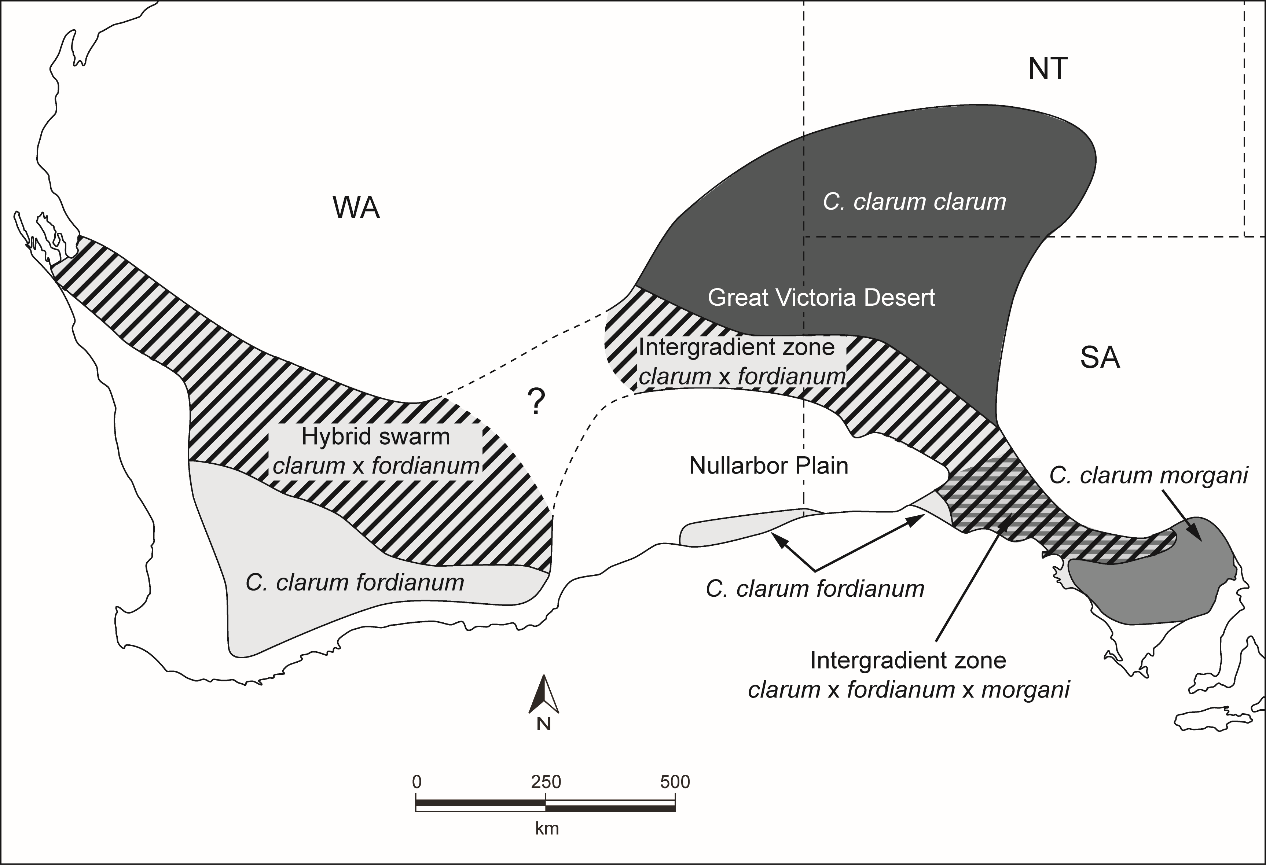


**Appendix Figure 2.** Results of DArT analysis. PCoA1 highlights separation of specimens of *C. castanotum* (purple dots) and *C. clarum* (green and shaded dots); registration numbers of all specimens in the ANWC are shown around each cluster. PCoA2 highlights separation of two clusters within *C. clarum*. See text for arguments that the specimens in the top right and lower right quadrants correspond to West_nuc_ and admixed East_nuc_/West_nuc_ nuclear genomes, respectively, as detected by STRUCTURE analysis (Figure 2). Abbreviations: NSW – New South Wales; SA – South Australia; WA – Western Australia.

**
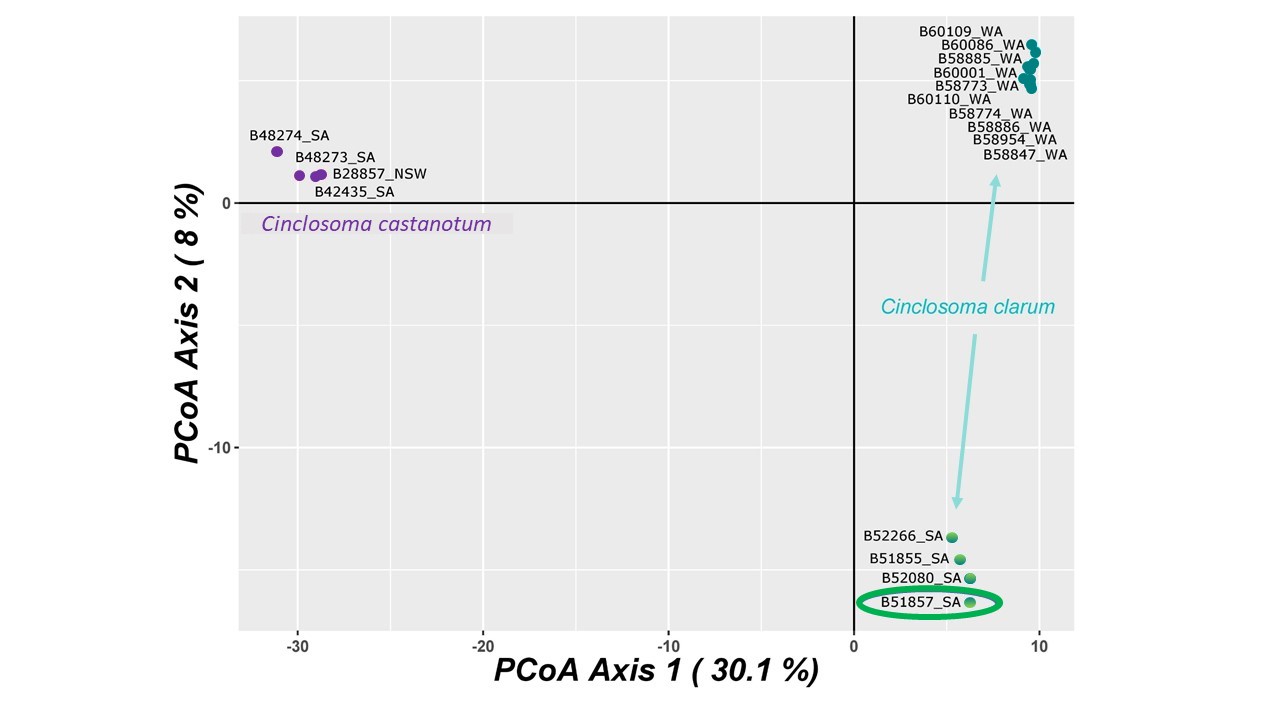
**

**Appendix Tables or Electronic Supplementary Material Tables**

**Appendix Table 1.** Details of all specimens (including year of collection) examined including mitochondrial DNA sequences from earlier papers (Toon *et al*., 2012, Dolman & Joseph, 2015), which were downloaded from Genbank (numbers beginning with prefixes KM or JN), or newly sequenced here (prefix MT). For each of the latter, the registration number of the voucher specimen accompanying that sequence is indicated. Newly sequenced samples lodged in GenBank have MT prefixes Australian Biological Tissue Collection (ABTC) numbers refer to cryofrozen tissues samples from the corresponding South Australian Museum, Adelaide (SAMA) voucher specimen. Abbreviated locality details are given, complete details being available from the Atlas of Living Australia ([www.ala.org](http://www.ala.org)), the indicated collections or earlier papers (Toon *et al*. 2012, Dolman & Joseph, 2015). Other abbreviations: ANWC – Australian National Wildlife Collection, CSIRO, Canberra; WAM - Western Australian Museum, Perth; RH – rockhole; HS – homestead; km -kilometres. Localities are abbreviated as follows: approximately 30 kilometres southeast of Mount Hope is given as ~30 km SE Mt Hope.

| Clade and Specimen | Species | Locality, Year of Collection |
| --- | --- | --- |
| **West_MT_** |  |  |
| MT296793 = ANWC B58886 | *Cinclosoma clarum* | 4 km S Jaurdi HS, 2017 |
| WAM A19681 | *Cinclosoma clarum* | Middini Beach, 1985 |
| WAM A30692 | *Cinclosoma clarum* | Gracefield, 1904 |
| WAM A13315 | *Cinclosoma clarum* | Burbidge, 1968 |
| WAM A17962 | *Cinclosoma clarum* | McDermid Rock, 1980 |
| KM280414.1 = ANWC B 51857 | *Cinclosoma clarum* | ~130 km N Cook, 2007 |
| WAM A30696 | *Cinclosoma clarum* | Cranbrook, probably 1904 |
| MT296791 = ANWC B60001 | *Cinclosoma clarum* | 10 km SE Fraser Range HS, 2017 |
| WAM A18168 | *Cinclosoma clarum* | Lake Barlee HS, 1983 |
| WAM A13309 | *Cinclosoma clarum* | 30 m S Yalgoo, 1966 |
| MT296795 = ANWC B58847 | *Cinclosoma clarum* | Credo Station Caravan dam, 2017 |
| MT296794 = ANWC B58885 | *Cinclosoma clarum* | 4 km S Jaurdi Homestead, 2017 |
| WAM A13243 | *Cinclosoma clarum* | Nevoria, 1965 |
| WAM A30688 | *Cinclosoma clarum* | Lake Dundas, 1905 |
| KM280403.1 = ANWC B33368 | *Cinclosoma clarum* | Tamala Station S Shark Bay, 2002 |
| WAM A13266 | *Cinclosoma clarum* | Mt Holland, 1966 |
| WAM A30691 | *Cinclosoma clarum* | Wongan Hills, 1903 |
| WAM A13298 | *Cinclosoma clarum* | 70 km N Kalgoorlie, 1967 |
| WAM A13281 | *Cinclosoma clarum* | Tamala, 1969 |
| WAM A30693 | *Cinclosoma clarum* | Gracefield, 1904 |
| WAM A30698 | *Cinclosoma clarum* | Cranbrook, probably 1904 |
| WAM A30694 | *Cinclosoma clarum* | Cranbrook, probably 1904 |
| WAM A13262 | *Cinclosoma clarum* | Tamala, 1971 |
| WAM A16798 | *Cinclosoma clarum* | Boondine Hill, 1978 |
| WAM A13257 | *Cinclosoma clarum* | Burbidge, 1965 |
| WAM A13252 | *Cinclosoma clarum* | 30km SE Yalgoo, 1964 |
| WAM A13263 | *Cinclosoma clarum* | 39 km E Meadow Homestead, 1965 |
| WAM A13242 | *Cinclosoma clarum* | Tamala, 1964 |
| WAM A13246 | *Cinclosoma clarum* | Tamala, 1965 |
| WAM A15139 | *Cinclosoma clarum* | Paynes Find, 1975 |
| WAM A15137 | *Cinclosoma clarum* | Mt Gibson, 1975 |
| KM280408.1 = ANWC B49654 | *Cinclosoma castanotum* | ~30 km SE Mt Hope, 1998 |
| KM280407.1= ANWC B49644 | *Cinclosoma castanotum* | ~30 km SE Mt Hope, 1998 |
| KM280406.1 = ANWC B48274 | *Cinclosoma castanotum* | 26 km N Arkaroola Station, 1996 |
| KM280401.1 = ANWC B28857 | *Cinclosoma castanotum* | ~3 km NW Lake Cargelligo, 1999 |
| JN620453.1 = ABTC 02669 = SAMA B47977 | *Cinclosoma castanotum* | Brookfield, 1993 |
| KM280404.1 = ANWC B 42435 | *Cinclosoma castanotum* | Goondooloo, 31 km W Halidon, 1989 |
| ANWC B38006 | *Cinclosoma castanotum* | 10 km SW Wynarka, 1983 |
| JN620451.1 = SAMA B56156 | *Cinclosoma castanotum* | Gluepot, 2009 |
| KM280402.1 = ANWC 28858 | *Cinclosoma castanotum* | ~30 km SE Mt Hope, 1999 |
| ANWC B11033 | *Cinclosoma castanotum* | Near Mt Hack, Flinders Ranges, 1967 |
| KM280405.1 = ANWC B48273 | *Cinclosoma castanotum* | 26 km N Arkaroola Station, 1996 |
| **East_MT_ Clade** |  |  |
| WAM A17964 | *Cinclosoma clarum* | 13 km N Junana Rock, 1983 |
| JN620454.1 = ABTC 73762 = SAMA B49118 | *Cinclosoma clarum* | 12.6 km NNE Lambina HS, 1999 |
| WAM A19680 | *Cinclosoma clarum* | Middini Beach, 1985 |
| WAM A13273 | *Cinclosoma clarum* | 97 km N Cook, 1969 |
| SAMA B37654 | *Cinclosoma clarum* | Maralinga Tjarutja, 1983 |
| WAM A13260 | *Cinclosoma clarum* | 20 km SE Cheesmans Peak, 1968 |
| WAM A15138 | *Cinclosoma clarum* | 60 km N Kalgoorlie, 1975 |
| JN620423.1 = ANWC B54296 | *Cinclosoma marginatum* of hybrid descent with *C. clarum* mtDNA | Neale Junction, 2008. Discussed in Toon *et al*. 2012 |
| KM280429.1 = SAMA B55864 | *Cinclosoma clarum* | 32 km W Yalata, 2007 |
| SAMA B32119 | *Cinclosoma clarum* | Bookaloo, 1979 |
| KM280420.1 = ANWC B52080 | *Cinclosoma clarum* | Irish Well, 2007 |
| MT296796 = ANWC B58774 | *Cinclosoma clarum* | 91 km N Norseman, 2017 |
| KM280427.1 = ANWC B54232 | *Cinclosoma clarum* | 48.6 km E Ilkurlka, 2008 |
| SAMA B58006 | *Cinclosoma clarum* | Oak Valley, 2006 |
| SAMA B58003 | *Cinclosoma clarum* | 13 m NE Kimba, 1974 |
| MT296788 = ANWC B60110 | *Cinclosoma clarum* | ~30 Km S Mundrabilla HS, 2017 |
| WAM A13416 | *Cinclosoma clarum* | Sir Frederick Range, 1973 |
| SAMA B58007 | *Cinclosoma clarum* | Poochera, 1990 |
| MT296792 = ANWC B58954 | *Cinclosoma clarum* | ~40 Km W Norseman, 2017 |
| SAMA B07704 | *Cinclosoma clarum* | Donald's Plains, 1902 |
| SAMA B54283 | *Cinclosoma clarum* | Donald's Plains, 1912 |
| KM280417.1 = ANWC B52039 | *Cinclosoma clarum* | 12.2 km S Vokes Hill Corner, 2007 |
| ANWC B40326 | *Cinclosoma clarum* | Sinclair Gap, 1985 |
| SAMA B31224 | *Cinclosoma clarum* | Secret Rocks, 1977 |
| KM280428.1 = ANWC B54234 | *Cinclosoma clarum* | 56.6 km E Ilkurlka, 2008 |
| MT296797 = ANWC B58773 | *Cinclosoma clarum* | 91 km N Norseman, 2017 |
| WAM A13307 | *Cinclosoma clarum* | Menzies, 1966 |
| WAM A13277 | *Cinclosoma clarum* | Coolgardie, 1970 |
| KM280415.1 = ANWC B51955 | *Cinclosoma clarum* | 5.4 km E Vokes Hill Corner, 2007 |
| SAMA B23064 | *Cinclosoma clarum* | 23 miles SW Iron Knob, 1925 |
| KM280409.1 = ANSP 22722 | *Cinclosoma clarum* | Yardea, Gawler Ranges, 2001 |
| KM280425.1 = ANWC 54218 | *Cinclosoma clarum* | 96.5 km E Ilkurlka, 2008 |
| KM280422.1 = ANWC B52089 | *Cinclosoma clarum* | 6 km N Irish Well, 2007 |
| KM280413.1 = ANWC B51855 | *Cinclosoma clarum* | ~100 km N Cook, 2007 |
| MT296790 = ANWC B60086 | *Cinclosoma clarum* | South from Madura, 2017 |
| KM280424.1 = ANWC B54215 | *Cinclosoma clarum* | 91.8 km E Ilkurlka, 2008 |
| KM280412.1 = ANWC B51838 | *Cinclosoma clarum* | 91 km N Cook, 2007 |
| KM280419.1 = ANWC B52041 | *Cinclosoma clarum* | 18.1 km S Vokes Hill Corner, 2007 |
| KM280410.1 = ANSP 22730 | *Cinclosoma clarum* | Yardea, Gawler Ranges, 2001 |
| SAMA B27521 | *Cinclosoma clarum* | 46 miles N Cowell, 1965 |
| SAMA B27520 | *Cinclosoma clarum* | 46 miles N Cowell, 1965 |
| MT296789 = ANWC B60109 | *Cinclosoma clarum* | ~30 km S Mundrabilla HS, 2017 |
| KM280411.1 = ANSP 22732 | *Cinclosoma clarum* | Yardea, Gawler Ranges, 2001 |
| WAM A13314 | *Cinclosoma clarum* | Eucla, 1970 |
| WAM A13272 | *Cinclosoma clarum* | 28km NE Maralinga, 1970 |
| WAM A13259 | *Cinclosoma clarum* | 1 km N Eucla, 1966 |
| KM280423.1 = ANWC B52266 | *Cinclosoma clarum* | 86.6 km NNE Ceduna, 2007 |
| SAMA B31225 | *Cinclosoma clarum* | Secret Rocks, 1977 |
| SAMA B23063 | *Cinclosoma clarum* | 23 miles SW Iron Knob, 1925 |
| ANWC B40327 | *Cinclosoma clarum* | Sinclair Gap, 1985 |
| WAM A13311 | *Cinclosoma clarum* | Balladonia, 1968 |
| SAMA B57995 | *Cinclosoma clarum* | 100 km N Cook, 2007 |
| KM280418.1 = ANWC B52040 | *Cinclosoma clarum* | 12.2 km S Vokes Hill Corner, 2007 |
| WAM A13265 | *Cinclosoma clarum* | 4 km W Callion, 1965 |
| ANWC B58008 | *Cinclosoma clarum* | 55 S Vokes Hill, 2007 |
| KM280421.1 = ANWC B52088 | *Cinclosoma clarum* | 6 km NE Irish Well, 2007 |
| ANWC B46039 | *Cinclosoma clarum* | Mitcherie RH, 1987 |
| WAM A13280 | *Cinclosoma clarum* | Vokes Hill Junction, 1969 |
| SAMA B55332 | *Cinclosoma clarum* | WNW Blowout Trig, 2005 |
| KM280416.1 = ANWC B51956 | *Cinclosoma clarum* | 5.4 km E Vokes Hill Corner, 2007 |
| KM280426.1 = ANWC B54225 | *Cinclosoma clarum* | 70.4 km E Ilkurlka, 2008 |
| ANWC B31662 | *Cinclosoma clarum* | 160 km N Cook, 2007 |
| ANWC B55919 | *Cinclosoma clarum* | E Colona, 2007 |
| WAM A13250 | *Cinclosoma clarum* | Eucla, 1967 |
| ANWC B27057 | *Ptilorrhoa caerulescens* | Kokoda environs, 1987 |
| ANWC B29240 | *Cinclosoma punctatum* | Yadboro State Forest, 2001 |

**Appendix Table 2.** Details of specimens studied and mitochondrial genome reconstructions. All are of *Cinclosoma* *clarum* except the last six which are other species of *Cinclosoma* as shown or from its sister group *Ptilorrhoa* (*P. caerulescens*). The 24 specimens selected for SNP analysis are *C. clarum* n = 18 (shown with *); *C. castanotum* (n = 4), *C. punctatum* (n = 1), and *Ptilorrhoa caerulescens* (n = 1) (see Appendix Table 3 for sequencing statistics). Localities of the 18 *C. clarum* and four *C. castanotum* so selected are shown with thick grey borders in Figure 2 of the main text. ‘Specimen’ shows each specimen’s unique museum catalogue number. Abbreviations as in Appendix Table 1. ‘Length’ is the total reconstructed length of the mitogenome aligned against the *T. guttata* reference mitochondrial genome. ‘Completion (%) is the percentage of called A, T, C or G bases within the reconstructed length. ‘Reference’ specifies whether the original reconstruction was with respect to the *T. guttata* reference genome or to the reconstructed mitogenome of *C. clarum* specimen SAMA B55864. ‘Manual’ indicates whether an attempt to further complete the reconstruction via manual inspection of the alignment was made. Reconstruction was not attempted for specimens WAM A30695 and WAM A19017 due to failed library construction (post-alignment estimates of fragment size were both 22 base pairs) and reconstruction was 16% complete for specimen WAM A30697. These specimens date from 1904, 1937, and 1903, respectively, potentially explaining the poor data quality and all three were excluded from further analysis. Abbreviation NA – not available, applies to these three specimens.

| **Specimen** | **Year of Collection** | **Length** | **Completion (%)** | **Reference** | **Manual** |
| --- | --- | --- | --- | --- | --- |
| WAM A13242 | 1964 | 16824 | 99.54 | B55864 | Yes |
| WAM A13243 | 1965 | 16824 | 100 | B55864 | Yes |
| WAM A13246* | 1964 | 16824 | 100 | B55864 | No |
| WAM A13250* | 1967 | 16824 | 100 | *T. guttata* | No |
| WAM A13252 | 1964 | 16824 | 100 | *T. guttata* | No |
| WAM A13257 | 1965 | 16824 | 99.92 | B55864 | No |
| WAM A13259 | 1966 | 16825 | 100 | *T. guttata* | Yes |
| WAM A13260 | 1968 | 16824 | 100 | *T. guttata* | No |
| WAM A13262 | 1971 | 16824 | 99.91 | B55864 | No |
| WAM A13263* | 1965 | 16824 | 100 | B55864 | No |
| WAM A13265 | 1965 | 16824 | 100 | *T. guttata* | No |
| WAM A13266 | 1966 | 16825 | 100 | *T. guttata* | No |
| WAM A13272 | 1966 | 16824 | 99.71 | B55864 | No |
| WAM A13273 | 1969 | 16823 | 100 | *T. guttata* | No |
| WAM A13277* | 1970 | 16823 | 100 | B55864 | No |
| WAM A13280 | 1969 | 16824 | 100 | *T. guttata* | No |
| WAM A13281 | 1969 | 16824 | 95.19 | B55864 | Yes |
| WAM A13298* | 1967 | 16824 | 100 | B55864 | No |
| WAM A13307 | 1966 | 16823 | 99.85 | B55864 | No |
| WAM A13309 | 1966 | 16824 | 100 | B55864 | No |
| WAM A13311 | 1968 | 16824 | 100 | B55864 | No |
| WAM A13314 | 1970 | 16825 | 99.77 | B55864 | No |
| WAM A13315 | 1968 | 16824 | 100 | B55864 | Yes |
| WAM A13416 | 1973 | 16824 | 99.74 | B55864 | No |
| WAM A15137* | 1975 | 16824 | 100 | B55864 | No |
| WAM A15138* | 1975 | 16824 | 100 | B55864 | No |
| WAM A15139 | 1975 | 16824 | 99.99 | B55864 | Yes |
| WAM A16798 | 1978 | 16824 | 100 | B55864 | No |
| WAM A17962* | 1980 | 16824 | 100 | B55864 | No |
| WAM A17964 | 1983 | 16824 | 100 | B55864 | No |
| WAM A18168* | 1983 | 16824 | 100 | B55864 | No |
| WAM A19017 | Failed | Failed | Failed | NA | NA |
| WAM A19680* | 1985 | 16824 | 100 | B55864 | No |
| WAM A19681* | 1985 | 16824 | 100 | B55864 | No |
| WAM A30688 | 1905 | 16824 | 99.77 | B55864 | No |
| WAM A30691 | 1903 | 16824 | 96.08 | B55864 | No |
| WAM A30692 | 1904 | 16824 | 97.75 | B55864 | No |
| WAM A30693 | 1904 | 16824 | 90.04 | B55864 | No |
| WAM A30694 | 1904 | 16824 | 99.93 | B55864 | No |
| WAM A30695 | Failed | Failed | Failed | NA | NA |
| WAM A30696 | 1904 | 16824 | 82.2 | B55864 | No |
| WAM A30697 | Failed | Failed | Failed | NA | NA |
| WAM A30698 | 1904 | 16824 | 98.69 | B55864 | No |
| SAMA B07704 | 1902 | 16827 | 99.98 | B55864 | Yes |
| SAMA B23063* | 1925 | 16824 | 100 | B55864 | No |
| SAMA B23064* | 1925 | 16825 | 100 | B55864 | No |
| SAMA B27520 | 1965 | 16824 | 100 | *T. guttata* | No |
| SAMA B27521 | 1965 | 16824 | 99.93 | B55864 | No |
| SAMA B31224* | 1977 | 16824 | 100 | B55864 | No |
| SAMA B31225 | 1977 | 16824 | 100 | *T. guttata* | No |
| SAMA B31662 | 2007 | 16824 | 100 | B55864 | No |
| SAMA B32119 | 1979 | 16824 | 100 | *T. guttata* | No |
| ANWC B33368*  clarum WEST Shark Bay | 2002 | 16824 | 100 | B55864 | No |
| SAMA B37654 | 1983 | 16824 | 100 | *T. guttata* | No |
| ANWC B40326 | 1985 | 16825 | 100 | B55864 | No |
| ANWC B40327 | 1985 | 16824 | 100 | B55864 | No |
| SAMA B46039 | 1987 | 16824 | 100 | *T. guttata* | No |
| SAMA B54283 | 1912 | 16824 | 99.93 | B55864 | No |
| SAMA B55332* | 2005 | 16824 | 100 | B55864 | No |
| SAMA B55864 | 2007 | 16824 | 100 | *T. guttata* | No |
| SAMA B55919 | 2007 | 16824 | 100 | *T. guttata* | No |
| SAMA B57995* | 2007 | 16824 | 100 | B55864 | No |
| SAMA B58003* | 1974 | 16824 | 100 | B55864 | No |
| SAMA B58006 | 2006 | 16839 | 100 | *T. guttata* | No |
| SAMA B58007 | 1990 | 16824 | 100 | *T. guttata* | No |
| SAMA B58008 | 2007 | 16824 | 100 | *T. guttata* | No |
| ANWC B38006 *castanotum* | 1983 | 16823 | 100 | B55864 | No |
| ANWC B42435 *castanotum* | 1989 | 16823 | 100 | B55864 | No |
| ANWC B48274 *castanotum* | 1996 | 16823 | 100 | B55864 | No |
| ANWC B11033 *castanotum* | 1967 | 16823 | 100 | B55864 | No |
| ANWC B29240 *punctatum* | 2001 | 16858 | 100 | B55864 | No |
| ANWC B27057 *P. caerulescens* | 1987 | 16887 | 100 | B55864 | No |

**Appendix Table 3.** Sequencing statistics for specimens assayed to higher coverage. All are of quailthrush *Cinclosoma* spp. as indicated except one from the sister genus *Ptilorrhoa* (*P. caerulescens*). ‘Specimen’ shows each specimen’s unique museum catalogue number. Abbreviations as in Appendix Table 1.

| Taxon | Specimen | Read Length (bp) | Mean Coverage (%) | High Quality Read Alignments (%) | Duplicates (%) | Median Insert Size |
| --- | --- | --- | --- | --- | --- | --- |
| *C. clarum* | WAM A13246 | 109118947 | 5.924849 | 67.617418 | 0.776 | 89 |
| *C. clarum* | WAM A13250 | 61245786 | 3.854113 | 69.437479 | 0.5179 | 101 |
| *C. clarum* | WAM A13263 | 29563561 | 1.610448 | 67.759165 | 0.28 | 89 |
| *C. clarum* | WAM A13277 | 43273130 | 2.321088 | 67.539441 | 0.5963 | 87 |
| *C. clarum* | WAM A13298 | 27745579 | 1.36565 | 66.369803 | 0.2484 | 81 |
| *C. clarum* | WAM A15137 | 56005383 | 3.894429 | 71.259632 | 0.6236 | 111 |
| *C. clarum* | WAM A15138 | 35238607 | 1.945669 | 68.240444 | 0.3002 | 89 |
| *C. clarum* | WAM A17962 | 59439639 | 3.842465 | 69.838161 | 0.5356 | 106 |
| *C. clarum* | WAM A18168 | 53724805 | 3.568703 | 70.731918 | 0.632 | 107 |
| *C. clarum* | WAM A19680 | 58951337 | 4.18827 | 71.512761 | 0.5619 | 115 |
| *C. clarum* | WAM A19681 | 50961102 | 3.543723 | 71.429209 | 0.4568 | 113 |
| *C. castanotum* | ANWC B11033 | 17323937 | 1.305844 | 72.305169 | 0.7337 | 124 |
| *C. clarum* | SAMA B23063 | 41781932 | 2.15777 | 67.60679 | 0.4687 | 86 |
| *C. clarum* | SAMA B23064 | 76836903 | 4.215965 | 66.041058 | 1.4876 | 95 |
| *P. caerulescens* | ANWC B27057 | 20454892 | 1.341752 | 68.018697 | 0.9207 | 109 |
| *C. punctatum* | ANWC B29240 | 61139019 | 1.419179 | 48.076777 | 5.2867 | 43 |
| *C. clarum* | SAMA B31224 | 35911501 | 2.224419 | 69.436048 | 0.5141 | 100 |
| *C. clarum* | ANWC B33368 | 29385228 | 2.380122 | 68.310853 | 0.8236 | 165 |
| *C. castanotum* | ANWC B38006 | 51396517 | 2.910149 | 62.077325 | 4.1017 | 114 |
| *C. castanotum* | ANWC B42435 | 17922179 | 1.216882 | 67.720633 | 1.1833 | 120 |
| *C. castanotum* | ANWC B48274 | 28666468 | 1.563396 | 60.80813 | 1.5795 | 107 |
| *C. clarum* | SAMA B55332 | 33570073 | 2.740961 | 72.198794 | 0.4792 | 141 |
| *C. clarum* | SAMA B57995 | 57117261 | 3.830954 | 68.844254 | 0.4548 | 113 |
| *C. clarum* | SAMA B58003 | 68579733 | 4.003641 | 68.76571 | 0.6055 | 97 |
